# Impact of template denaturation prior to whole genome amplification on gene detection in high GC-content species, *Burkholderia mallei* and *B. pseudomallei*

**DOI:** 10.1186/s13104-024-06717-8

**Published:** 2024-03-12

**Authors:** Chris R. Taitt, Tomasz A. Leski, Jaimee R. Compton, Amy Chen, Kimberly L. Berk, Robert W. Dorsey, Shanmuga Sozhamannan, Dianne L. Dutt, Gary J. Vora

**Affiliations:** 1 Nova Research Inc., Alexandria, VA 22308 USA; 2 Center for Biomolecular Science & Engineering, US Naval Research Laboratory, Washington, DC USA; 3 Karle’s Fellow, US Naval Research Laboratory, Washington, DC USA; 4 US Army Combat Capabilities Development Command—Chemical Biological Center, Aberdeen Proving Ground, MD USA; 5 Defense Biological Product Assurance Office, Joint Program Executive Office for Chemical, Biological, Radiological and Nuclear Defense (JPEO-CBRND), Frederick, MD USA; 6 Joint Research and Development, Inc., Stafford, VA USA; 7 Defense Threat Reduction Agency, Joint Science and Technology Office, Ft. Belvoir, VA USA

**Keywords:** Antimicrobial resistance, Resistome, Extended-spectrum β-lactamase, ESBL, Select agents

## Abstract

**Objective:**

In this study, we sought to determine the types and prevalence of antimicrobial resistance determinants (ARDs) in *Burkholderia* spp. strains using the Antimicrobial Resistance Determinant Microarray (ARDM).

**Results:**

Whole genome amplicons from 22 *B. mallei* (BM) and 37 *B. pseudomallei* (BP) isolates were tested for > 500 ARDs using ARDM v.3.1. ARDM detected the following *Burkholderia* spp.-derived genes, *aac(6)*, *bla*_BP/MBL-3_, *blaA*_BPS_, *penA*-*BP*, and *qacE*, in both BM and BP while *bla*_BP/MBL-1_, *macB*, *bla*_OXA-42/43_ and *penA*-*BC* were observed in BP only. The method of denaturing template for whole genome amplification greatly affected the numbers and types of genes detected by the ARDM. *Bla*_TEM_ was detected in nearly a third of BM and BP amplicons derived from thermally, but not chemically denatured templates. *Bla*_TEM_ results were confirmed by PCR, with 81% concordance between methods. Sequences from 414-nt PCR amplicons (13 preparations) were 100% identical to the *Klebsiella pneumoniae* reference gene. Although *bla*_TEM_ sequences have been observed in *B. glumae*, *B. cepacia*, and other undefined *Burkholderia* strains, this is the first report of such sequences in BM/BP*/B. thailandensis* (BT) clade. These results highlight the importance of sample preparation in achieving adequate genome coverage in methods requiring untargeted amplification before analysis.

**Supplementary Information:**

The online version contains supplementary material available at 10.1186/s13104-024-06717-8.

## Introduction

Antimicrobial resistance (AMR) is among the World Health Organization’s most important global public health threats and is attributed with 1.27 million deaths in 2019 [1]. While significant efforts are being made to address and mitigate AMR in the public health sector [2–4], the mechanisms and prevalence of AMR in Tier 1 Select Agents and their near neighbors are not as comprehensively defined. Notably, infections with *B. pseudomallei* (BP, melioidosis) and *B. mallei* (BM, glanders) are estimated to affect up to 165,000 humans and hundreds to thousands of equids each year, respectively [5–7]. While both diseases have mortality rates of 90–95% in untreated humans, even the current two-phase therapeutic guidelines (2–8 weeks intravenous antimicrobials + 3–6 months of oral antimicrobials) may fail in up to 40% of cases [5, 8, 9]. Myriad intrinsic antimicrobial resistance (AMR) mechanisms including penicillin-binding proteins, PenA β-lactamases, drug efflux pumps, unusual lipopolysaccharide structure, altered target sites, target overproduction, and intracellular pathogen localization contribute to these therapeutic challenges [10, 11]. Furthermore, genome complexity/plasticity and reports of engineered resistance [12, 13] increase the potential for emergence and spread of resistant strains with therapeutic options more limited than those in current use [8, 9, 14].

This work is an extension to a previously published survey of Category A Select Agents and exempt strains for horizontally and vertically transferred AMR determinants [15]. Here, we use the Antimicrobial Resistance Determinant Microarray (ARDM) v.3.1 for broad spectrum screening of > 500 AMR determinants in 22 BM and 37 BP strains, with PCR as an orthogonal detection method.

## Methods

Purified DNA preparations from 22 BM and 37 BP Unified Culture Collection (UCC) strains were obtained through the Genomic Repository Program at DEVCOM CBC of the US Defense Biological Product Assurance Office (DBPAO), Frederick, MD, USA. Whole genome amplification (WGA) was performed on each sample using Illustra GenomiPhi HY kits (GE Healthcare, USA) essentially as described by the manufacturer’s instructions, using 10–25 ng of starting material and amplifying for 2 h at 30 ℃. Because of *Burkholderia*’s high GC content, two approaches for WGA template denaturation were compared. Thermal denaturation involved template incubation for 5 min at 95 ℃ and 3 min on ice before WGA (“thermal amplicons”). Chemical denaturation involved template treatment for 3 min with Buffer D1 (REPLI-g Mini kit; Qiagen, USA, 1:1 volume:volume ratio), followed by two volumes of Buffer N1 (same kit) before WGA (“chemical amplicons”). Amplification time was set to 2 h to allow sufficient amplicon formation while preventing non-specific background amplification.

Equivalent amounts (3.2 µg) of thermal or chemical amplicons were fragmented and labeled using Bionick DNA-Labeling System (ThermoFisher). Fragmented, biotinylated amplicons were then applied without purification to the ARDM v.3.1 (Customarray, USA), and hybridized overnight at 60 ℃ as previously described [15, 16]. Hybridized microarrays were processed, labeled, and interrogated using the Electrasense Reader (Customarray) and previously established positive/negative thresholds [15]. Burkholderiales-specific ARDM content is found in Additional File 1.

PCR assays targeting *bla*_TEM_ and five BM/BP-derived genes were used to confirm their presence/absence in a subset of thermal and chemical amplicons (Additional File 2). PCR amplification was assessed via electrophoresis (FlashGel, Lonza, USA). Published NCBI sequences with ≥ 95% sequence identity were used as the gold standard for sensitivity/specificity.

## Results and discussion

Samples used in the ARDM and PCR analyses were generated via phi29-based WGA, a robust and reliable method to obtain large quantities of high-fidelity amplicons with near-complete genome representation. However, several groups have observed GC content-based biases using this method [17–20] and, in a previous study, we postulated that minor differences in microarray results between thermal and chemical amplicons may have been due to differences in GC content between the specific genes and the host genome [16]. Based on *Burkholderia’s* high GC content (61–68%), its genome plasticity, and the potential for genomic islands arising from other species by horizontal gene transfer [21–23], we performed WGA using templates denatured thermally or chemically to assess ARDM performance.

In general, chemical (alkali) denaturation of BM/BP templates provided higher yields after WGA than thermal denaturation (Mann–Whitney, *p* < 0.001; Additional File 3). To prevent over- or under-fragmentation and labeling in subsequent steps, both chemical and thermal amplicons were normalized to 3.2 µg before processing with the Bionick kit; labeled, fragmented amplicons were not re-quantified prior to ARDM analysis, however.

### Detection of BM/BP-derived genes (sensitivity/specificity)

ARDM and PCR results for each sample, as well as accession numbers and presence/absence of each BM/BP-derived gene, are found in Additional File 4.

Using thermal amplicons, ARDM analysis detected only four BM/BP-derived genes (*bla*_BP/MBL-3_, *bla*_OXA-42/43_, *qacE*, and *penA-BP*) in significant sample numbers, indicating this method’s overall poor sensitivity with this sample set (Table 1)*.* Sensitivities ranged from 0 to 100%, depending on the species and specific gene. In general, a higher proportion of ARDM-positives were observed in BM than in BP (χ^2^, *p* < 0.05); for example, sequences for *blaA*_BPS_ and *penA-BP* are found in both species but were detected at minimal levels in BP, yielding only 12% and 25% overall sensitivities, respectively. On the other hand, *bla*_BP/MBL-1_, *bla*_OXA-42/43_, and *macB*—found only in BP—were not observed in any BM strains tested, indicating 100% specificity for these genes.

**Table 1:** Overview of *Burkholderia*-derived genes and *bla*_TEM_ detected in BM/BP thermal and chemical amplicons

	Presence of sequences with ≥ 95% identity in NCBI		Thermal amplicons				Chemical amplicons				ARDM/PCR concordance	
	BM	BP	BM		BP		BM		BP		Thermal	Chemical
	n = 22	n = 37	ARDM	PCR	ARDM	PCR	ARDM	PCR	ARDM	PCR		
*aac(6)*	100%	100%	50% (10/20)	0% (0/8)	14% (5/37)		74% (14/19)	100% (12/12)	59% (20/34)	100% (35/35)	13%	66%
*bla*A_BPS_	100%	100%	35% (7/20)	0% (0/8)	0% (0/37)		47% (9/19)	100% (12/12)	47% (16/34)	57% (20/35)	33%	52%
*bla*_BP/MBL-1_	0	100%	0% (0/20)	0% (0/8)	5% (2/37)	0% (0/1)	0% (0/19)	0% (0/12)	71% (24/34)	97% (34/35)	92%	80%
*bla*_BP/MBL-3_	100%	100%	100% (20/20)	100% (11/11)	68% (25/37)	100% (1/1)	95% (18/19)	100% (12/12)	100% (34/34)	100% (35/35)	100%	100%
*bla*_OXA-42/43_	0%	100%	0% (0/20)	0% (0/8)	62% (23)	100% (1/1)	0% (0/19)	0% (0/12)	94% (32/34)	100% (35/35)	100%	95%
*bla*_TMB_^a^	0%	0%	0% (0/20)		0% (0/37)		0% (0/19)		0% (0/34)			
*bla*_VEB_^a^	0%	0%	0% (0/20)		0% (0/37)		0% (0/19)		0% (0/34)			
*macB*	0%	0%	0% (0/20)		11% (4/37)		0% (0/19)		85% (29/34)			
*penA-BP*	100%	100%	65% (13/20)	73% (8/11)	3% (1/37)	0% (0/1)	89% (17/19)	75% (9/12)	76% (26/34)	57% (20/35)	67%	68%
*penA-BC*^b^	0%	0%	0% (0/20)		0% (0/37)		0% (0/19)		12% (4/34)			
*qacE*	100%	100%	100% (20/20)		84% (31/37)		95% (18/19)		100% (34/34)			
*bla*_TEM_	0%	100%	35% (7/20)	45% (10/22)	35% (13/37)	14% (5/37)	0% (0/19)	0% (0/7)	0% (0/34)	0% (0/5)	77%	100%

^a^Sequence derived from *Achromobacter* sp. (Burkholderiales)

^b^Sequence, derived from *B. cepacia*, has 78% sequencing identity to *penA-BP* over 82% of the entire gene

Confirmatory PCRs detected only *bla*_BP/MBL-3_ and *penA-BP* in significant proportions of the limited number of the thermal amplicons tested; as expected, *bla*_OXA-42/43_ was not detected in BM strains (100% specificity). PCR assays for both *aac(6)* and *blaA*_BPS_ provided no positive results amongst thermal amplicons; high concordances between ARDM and PCR were observed for *bla*_BP/MBL-1_, *bla*_BP/MBL-3_, and *bla*_OXA-42/43_. However, for the thermal amplicon set, comparisons between ARDM and PCR may not be significant, given the small number of samples tested by PCR.

When chemical amplicons were used as samples, ARDM detected all BM/BP-derived genes except *qac* at significantly higher proportions when compared with matched thermal amplicons (*n* = 51 pairs; McNemar, *p* < 0.01, Table 2). ARDM sensitivities were therefore higher for chemical amplicons, ranging from 59 to 100% (Table 1). As with thermal amplicons, *bla*_BP/MBL-1_, *bla*_OXA-42/43_, and *macB* were detected only in BP (100% specificity for each). PCR performed better than ARDM at detecting *aac(6)*, *blaA*_BPS_, and *bla*_BP/MBL-1_ in chemical amplicons (*n* = 44 matched samples for *aac(6)* and *blaA*_BPS_, *n* = 32 BP only for *bla*_BP/MBL-1_; McNemar’s test, *p* < 0.02) but the opposite was true for *penA-BP* (*n* = 44, McNemar’s test, *p* = 0.016).

**Table 2:** Detection of BM/BP-derived genes, *bla*_TEM_, and others in matched pairs of thermal/chemical samples (n = 51)

Denaturation	Thermal (−)/chemical (−)	Thermal ( +) /chemical ( +)	Thermal (−)/chemical ( +)	Thermal ( +)/chemical (−)	McNemar’s P
*aac(6)*	15	8	24	4	< 0.001
*bla*_BP/MBL-1_	27	1	23	–	< 0.001
*bla*_BP/MBL-3_	–	38	12	1	0.006
*blaA*_BPS_	23	1	23	4	< 0.001
*bla*_OXA-42/43_	19	20	12	–	0.001
*macB*	22	1	28	–	< 0.001
*penA-BP*	9	9	32	1	< 0.001
*qacE*	–	43	6	1	0.131
*bla*_TEM_	35	–	–	16	< 0.001
Other non-BM/BP genes (18 genes)	874	–	32	12	0.004
TOTAL	1024 (74%)	121 (9%)	192 (14%)	39 (3%)	

Insufficient numbers of assays for BM/BP-derived genes were performed with both sample populations for a statistically robust comparison of PCR performance between thermal and chemical amplicons. However, PCR assays tended to detect BM/BP-derived genes in a higher proportion of chemical amplicons than in thermal amplicons (better sensitivity) and with improved ARDM concordance. PCR specificities for *bla*_BP/MBL-1_, *bla*_OXA-42/43_, and *macB* (found only in BP) were 100% in both thermal and chemical amplicons.

### Detection of genes not derived from BM/BP

ARDM detected 19 non-BM/BP-derived genes in thermal (7 genes) and chemical (13 genes) amplicons, all potentially representing false positives. None were detected in both chemical and thermal amplicons from the same strain. Excluding *bla*_TEM_ (discussed below), these genes were detected more frequently in chemical versus thermal amplicons when comparing matched samples (McNemar, *p* = 0.004) but differences were not significant when comparing populations as a whole (χ^2^, *p* = 0.902). Interestingly, the GC contents of false-positive identifications from thermal amplicons were lower than those from chemical amplicons (t-test, *p* < 0.001; Mann–Whitney, *bla*_TEM_ included; *p* < 0.001; Additional File 5).

Detection of *bla*_TEM_ in nearly one-third of thermal amplicons via two orthogonal methods—but not in chemical amplicons by either—represents a unique observation, though ARDM-PCR concordance was relatively poor. PCR amplicons from thirteen *bla*_TEM_-positive (thermal amplicon) samples were purified and sequenced, and yielded sequences 100% identical to the ARDM *bla*_TEM_ reference gene (AF309824: 119..979; Table 3, top). To our knowledge, these observations represent the first time that *bla*_TEM_ has been detected in the BM/BP/BT group, though analogous sequences have been documented in strains from other *Burkholderia* clades ([24–29] and Table 3). A BLAST search of NCBI yielded nine records from *Burkholderia* spp. with > 99% identity to the purified amplicons over the entire 414-nt length (Table 3, bottom). While eight are partial sequences, the *B. glumae* and *B. cepacia* sequences are > 99% identical to the entire 861-nt *bla*_TEM-1_ reference gene.

**Table 3:**
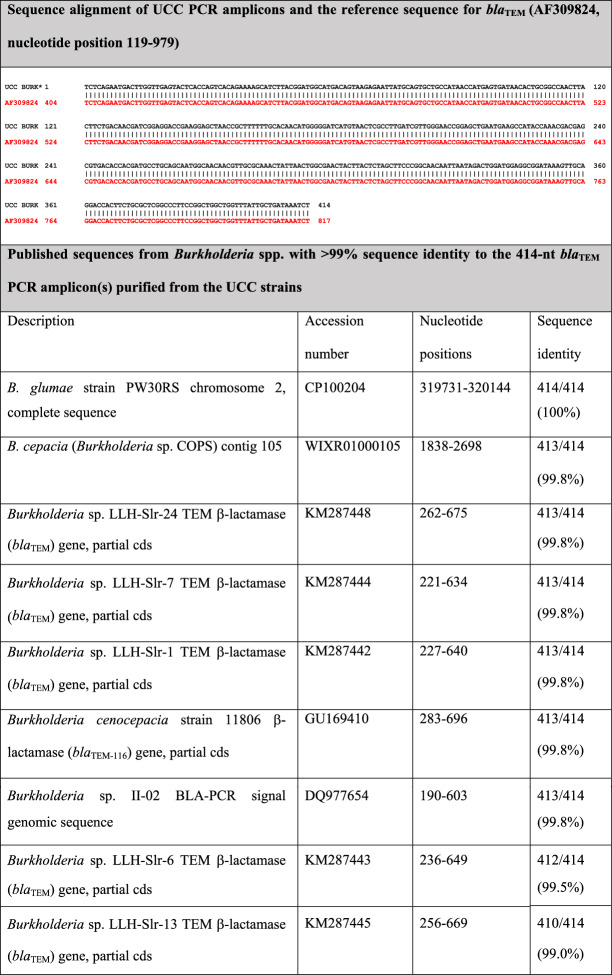
*Bla*_TEM_ PCR amplicon sequences from UCC strains (top) and other *Burkholderia* clades (bottom)

^*^UCC BURK represents (identical) amplicon sequences from the following UCC *Burkholderia* spp. strains: BM: BURK007, BURK010, BURK065, BURK066, BURK073, BURK077, BURK082, BURK119, BURK131; BP: BURK132, BURK133, BURK134, and BURK135. These sequences are available in NCBI as accession numbers OR815368 through OR815380. The reference sequence for *bla*_TEM_ (AF309824) is shown in red

This study’s differential detection of *bla*_TEM_ in thermal amplicons only may be due to template denaturation, the gene’s GC content, and/or its context within the genome(s). Here, *bla*_TEM_ and other genes with lower GC content were more frequently detected in thermal amplicons, whereas *Burkholderia* sp.-derived and other genes with GC > 50% tended to be detected more frequently in chemical amplicons. These results support observations that GC-rich regions are underrepresented in WGA amplicons from thermally denatured templates [18, 30, 31], though conflicting reports have also been published [17, 32]. While detection of *bla*_TEM_ in our thermal amplicons may be artifacts from enrichment of lower GC regions, we note that the genome context of the *B. glumae bla*_TEM_ gene (Table 3; CP100204) is within a chromosomal region with lower GC content, where the upstream six genes are > 95% identical in sequence to *Escherichia coli* analogs. It is possible that genome plasticity—documented to occur in *Burkholderia* spp. [33–38]—is responsible for horizontal transfer of this gene from a Gammaproteobacterium, *Neisseriaceae* spp., or one of the few other Betaproteobacteria species in which *bla*_TEM_ has been documented. We have not attempted to identify the full *bla*_TEM_ gene or its genome context within the strains tested here. It further remains to be seen whether the detected sequences are part of a complete *bla*_TEM_ gene that is actively transcribed and is capable of conferring a clinically relevant phenotype (e.g., resistance to ceftazidime and/or amoxicillin/clavulanate, used in acute and eradication phase therapies for melioidosis and glanders).

## Limitations

While useful to track movement of ARDs in epidemiological studies, the ARDM technology used here is unable to predict AMR phenotypes based on differences in transcriptional regulation or gene duplication. Furthermore, as ARDM probes are designed to detect ARD sequences conserved amongst multiple species, ARDM chips cannot detect small sequence differences affecting phenotype (e.g., *penA* mutations conferring ceftazidime resistance; [39]). However, these limitations are shared by any DNA-based technologies using relatively long sequences for hybridization-based detection. Newer technologies such as gene expression profiling—especially when combined with next generation sequencing approaches—can provide valuable information about both transcriptional levels and the presence of mutations, increasing the potential for phenotypic predictions. Untargeted sequencing technologies also have the potential to detect all known and suspected determinants—well above the current content (~ 500 genes) of the ARDM chips—provided the full genome is sufficiently represented.

Here, we observed significant differences in the numbers and types of genes detected based on the method of pre-WGA template denaturation. These and other WGA-induced artifacts can also affect other analytical methods requiring untargeted amplification before analysis, i.e., where sample size is limited, including sequencing [40–43]. While other efforts to sequence the same UCC strains have failed to document *bla*_TEM_ in the BM/BP/BT clade, the current study presents evidence to support its natural occurrence within the tested strains, and potentially in a wider context. Specifically, detection of the low-GC-content *bla*_TEM_ gene in nearly a third of thermal amplicons due to a fortuitous artifact of template denaturation before WGA (i.e., enrichment of AT-rich regions) may enable detection of other genes not easily identified, although the relatively poor ARDM/PCR concordance (77% in the thermal amplicons) should be explored further. Detection of *bla*_TEM_ in both BM and BP amplicons prepared on multiple days, in multiple preparations, and in multiple facilities suggest that positives were not due to contamination; other samples processed at the same time were all *bla*_TEM_-negative except where expected [15]. Finally, sequences from thirteen purified PCR amplicons were 100% identical to the *E. coli* reference gene over the full 414-nt amplicon length.

More research is needed to characterize the generally poor detection of BM/BP ARDs and low ARDM/PCR concordance in thermal versus chemical amplicons. Specifically, differences in representation of various regions of the genome between thermal and chemical amplicon populations may shed light on unidentified genes and regions of the genome previously missed due to requirements for large quantities of DNA obtainable only through amplification (e.g., sequencing and hybridization applications). Overall, this study suggests that use of both thermal and chemical template denaturation may enable detection of *bla*_TEM_ and other previously undetected genes in high or low GC backgrounds. Confirmation of *bla*_TEM_ presence and clinical importance will require full sequence and context determinations, while application of these observations to other high GC species will require a larger and more complex sample set.

### Supplementary Information

**Additional file 1. **Content of ARDM v.3.1 derived from Burkholeriales; Description—Antimicrobial resistance determinants represented on ARDM v.3.1 that are derived from species in Burkholderiales.

**Additional file 2. **PCR primers and conditions; Description—Primers (and cycling conditions) used for PCR confirmation of select BM/BP-derived determinants and *bla*_TEM._

**Additional file 3. **Distribution of yields from WGA; Description—Distribution curves of yields from WGA amplicons from thermally and chemically denatured templates.

**Additional file 4. **Results from ARDM and PCR; Format: Excel spreadsheet; Description—Strain descriptions, presence/absence of BM/BP-derived genes and *bla*_TEM_ within published genomes, detection of BM/BP-derived genes and *bla*_TEM_ via ARDM analysis and PCR.

**Additional file 5. **Putative false-positive (non-BM/BP-derived) genes detected by ARDM; Description—Prevalence of non-BM/BP-derived genes detected by ARDM analysis amongst thermal and chemical samples (Panel **A**) and distribution of GC contents of non-BM/BP-derived genes detected (Panel **B**).

## Data Availability

Supplemental data are found in the attached files; full ARDM v.3.1 content can be obtained from the US Defense Biological Product Assurance Office (DBPAO), Frederick, MD, USA. Nucleic acid materials can be obtained from the Unified Culture Collection (UCC) of DBPAO. Amplicon sequences are available in NCBI.

## Declarations

### Non-endorsement

References to non-federal entities or commercial products do not constitute or imply Department of Defense or U.S. Army endorsement of any company, product, or organization.
