# Supplementary material for: Impact of template denaturation prior to whole genome amplification on gene detection in high GC-content species, Burkholderia mallei and B. pseudomallei
Source: BMC Res Notes. 2024 Mar 12;17:70. doi: 10.1186/s13104-024-06717-8 (PMC10935807; doi:10.1186/s13104-024-06717-8)
Supplement: Supplementary file 1 — Additional file 1. Content of ARDM v.3.1 derived from Burkholeriales; Description—Antimicrobial resistance determinants represented on ARDM v.3.1 that are derived from species in Burkholderiales. [file 13104_2024_6717_MOESM1_ESM.pdf]

Additional file 1. Content of ARDM v.3.1 derived from Burkholderiales.

Gene	Protein encoded	NCBI accession number	Location	Sequence with >90% identity found in:	
				**B. mallei**	**B. pseudomallei**
aac(6)[1]	Putative aminoglycoside 6'-acetyltransferase	BX571966	349788-350261	√	√
*bla*A$_{BPS}$[2,3]	Class A β-lactamase	AF326770	1-888	√	√
bla$_{BP/MBL-1}$[4]	Metallo-β-lactamase	NC_006350	1811369-1812292		√
bla$_{BP/MBL-3}$[4]	Metallo-β-lactamase	NC_006350	3892896-3893540	√	√
bla$_{OXA-42/43}$[2,5]	Class D β-lactamase	AJ488303	1-810		√
bla$_{TMB}$[6]	Metallo-β-lactamase	FR771847	16-753		
bla$_{VEB}$[7]	Extended-spectrum β-lactamase	DQ393569	1955-2854		
macB[8]	Macrolide-specific efflux pump	BX571966	851202-853163		√
penA (*penA-BP*)[5]	Class A β-lactamase	AY032868	91-978	√	√
penA (*penA-BC*)[9]	Class A β-lactamase	U85041	1163-2017		
qacE[10]	Quaternary ammonium compound resistance protein	BX571965	2215194-2215532	√	√

Full ARDM v.3.1 content can be requested from DBPAO.

Notes on potential role in resistance, rationale for inclusion on ARDM v.3.1:

[1] Aac(6') homolog and variants found only in *Burkholderia* spp, including *B. pseudomallei*, *B. mallei*, and *B. oklahomensis*. Protein with 98.7% identity confers a 16-fold increase in MIC for tobramycin, though not gentamicin (to which *B. mallei* and a few *B. pseudomallei* strains are susceptible) [1, 2].

[2] Subset of β-lactamase genes used to discriminate between *B. mallei*, *B. pseudomallei*, *B. thailandensis*, and *B. cepacia* [3]. May contribute to broad-spectrum β-lactam non-susceptibility

[3] Contributes to resistance to cefepine, cefpirome (4th generation cephalosporins, not clinically relevant for melioidosis, glanders therapies), responsible for 2-fold increase in MIC for meropenem (may be clinically relevant) [4].

Distribution Statement A: Approved for public release: distribution is unlimited

[4] Potential to affect susceptibility to carbapenems

[5] Overexpression correlated with decreased ceftazidime susceptibility [5-7]

[6] Carbapenemase gene derived from *Achromobacter,* also found in Enterobacteriaceae [8, 9]; included in ARDM v.3.0

[7] Broadly distributed extended-spectrum β-lactamase [10]; reference sequence derived from *Achromobacter* sp. (Burkholderiales) [11]. Included in all previous versions of the ARDM (v.1, v.2, and v.3)

[8] One of multiple efflux pumps in *Burkholderia* spp. Not demonstrated to generate clinically significant resistance [12, 13].

[9] Sequence derived from *B. cepacia*, has 78% sequence identity to *penA-BP* over 82% of the entire gene.

[10] Potential to confer tolerance to disinfectants

REFERENCES:

1. Zhang, G.; Li, J.; Ai, G.; He, J.; Wang, C.W.; Feng, J. A new intrinsic aminoglycoside 6′-N-acetyltransferase subclass, AAC(6′)-III, in *Burkholderia pseudomallei*, *Burkholderia mallei* and *Burkholderia oklahomensis. J. Antimicrob. Chemother*. **2020**, *75*, 1352-1353.

2. Mandell, G.L.; Bennett, J.E.; Dolin, R. *Mandell, Douglas, and Bennett's Principles and Practice of Infectious Diseases*. Philadelphia: Churchill Livingstone, 2010.

3. Zakharova, I.; Teteryatnikova, N.; Toporkov, A.; Viktorov, D. Development of a multiplex PCR assay for the detection and differentiation of *Burkholderia pseudomallei*, *Burkholderia mallei*, *Burkholderia thailandensis*, and *Burkholderia cepacia* complex. *Acta Trop.* **2017**, *174*, 1-8.

4. Cheung, T.K.; Ho, P.L.; Woo, P.C.; Yuen, K.Y.; Chau, P.Y. Cloning and expression of class A beta-lactamase gene $blaA_{\mathrm{BPS}}$ in *Burkholderia pseudomallei. Antimicrob. Agents Chemother.* **2002**, *46*, 1132-5.

5. Niumsup, P.; Wuthiekanun, V. Cloning of the class D beta-lactamase gene from *Burkholderia pseudomallei* and studies on its expression in ceftazidime-susceptible and -resistant strains. *J. Antimicrob. Chemother.* **2002**, *50*, 445-455.

6. Chirakul, S.; Norris, M.H.; Pagdepanichkit, S.; Somprasong, N.; Randall, L.B.; Shirley, J.F.; Borlee, B.R.; Lomovskaya, O.; Tuanyok, A.; Scheweizer, H.P. Transcriptional and post-transcriptional regulation of PenA β-lactamase in acquired *Burkholderia pseudomallei* β-lactam resistance. *Sci. Rep.* **2018**, *8*, 10652.

7. Chirakul, S.; Somprasong, N.; Norris, M.H.; Withiekanun, V.; Chantratita, N.; Tuanyok, A.; Schweizer, H.P. *Burkholderia pseudomallei* acquired ceftazidime resistance due to gene duplication and amplification. *Int. J. Antimicrob. Agents* **2019**, *53*, 582-588.

8. Gauthier, L.; Dortet, L.; Jousset, A.B.; Mihaila, L.; Golse, N.; Naas, T.; Bonnin, R.A. Molecular characterization of plasmid-encoded Tripoli MBL 1 (TMB-1) in Enterobacteriaceae. *J. Antimicrob. Chemother*. **2019**, *74*, 42-47.

9. El Salabi, A.; Borra, P.S.; Toleman, M.A.; Samuelsen, Ø.; Walsh, T.R. Genetic and biochemical characterization of a novel metallo-β-lactamase, TMB-1, from an *Achromobacter xylosoxidans* strain isolated in Tripoli, Libya. *Antimicrob. Agents Chemother.* **2012**, *56*, 2241-5.

10. Poirel, L.; Naas, T.; Guibert, M.; Chaibi, E.B.; Labia, R.; Nordmann, P. Molecular and biochemical characterization of VEB-1, a novel class A extended-spectrum beta-lactamase encoded by an *Escherichia coli* integron gene. *Antimicrob. Agents Chemother.* **1999**, *43*, 573-81.

11. Neuwirth, C.; Freby, C.; Ogier-Desserrey, A.; Perez-Martin, S.; Houzel, A.; Péchinot, A.; Duez, J.M.; Huet, F.; Siebor, E. VEB-1 in *Achromobacter xylosoxidans* from cystic fibrosis patient, France. *Emerging Infect. Dis.* **2006**, *12*, 1737-9.

12. Rhodes, K.A.; Schweizer, H.P. Antibiotic resistance in *Burkholderia* species. *Drug Resistance Updates* **2016**, *28*, 82-90.

13. Schweizer, H.P. Mechanisms of antibiotic resistance in *Burkholderia pseudomallei*: implications for treatment of melioidosis. *Future microbiology* **2012**, *7*, 1389-1399.
