# Supplementary material for: Impact of template denaturation prior to whole genome amplification on gene detection in high GC-content species, Burkholderia mallei and B. pseudomallei
Source: BMC Res Notes. 2024 Mar 12;17:70. doi: 10.1186/s13104-024-06717-8 (PMC10935807; doi:10.1186/s13104-024-06717-8)
Supplement: Supplementary file 2 — Additional file 2. PCR primers and conditions; Description—Primers (and cycling conditions) used for PCR confirmation of select BM/BP-derived determinants and blaTEM. [file 13104_2024_6717_MOESM2_ESM.pdf]

Additional File 2. PCR Primers and conditions

Primers (and cycling conditions) used for PCR confirmation of select *B. mallei*-/*B. pseudomallei*-derived determinants and *bla*$_{TEM}$

Gene	Primer sequences	Amplicon size
bla$_{TEM}$*	TEM-F: CGCCGCATACACTATTCTCAGAATGA<br>TEM-R: ACGCTCACCGGCTCCAGATTTAT	445
aac(6)	aac(6)-burk-F2: CTTGCGGAAGAACACGACC<br>aac(6)-burk-R2: GATGACGTGAACGGTTGCG	237
bla$_{MBL-1}$	bla$_{MBL-1}$-F1: GAGCGGGATCTTCAGGTAGC<br>bla$_{MBL-1}$-R1: GACGGTCGAAGGCTTTTTCG	871
bla$_{MBL-3}$	bla$_{MBL-3}$-F1: AAAGTCACGCTCATTCCGGT<br>bla$_{MBL-3}$-R1: CAGGGCAGTGATAGACCTCG	388
blaA$_{BPS}$	blaBPS-F2: CCGTTTTGCAGCACATCCAA<br>blaBPS-R2: AGACGATGAACACGATCGGC	586
bla$_{OXA-42/43}$	bla$_{OXA-42}$-F1: CTGTCGAGCGCATTCGTTTT<br>bla$_{OXA-42}$-R1: ATTCCAATTGTTCGAGCGGC	472
*penA-BP*1	penA-B1-F1: GAATCATTCTCCGTTGCGCC<br>penA-B1-R1: TTGTCGCTGTACTGAAGCGT	411

* PCR primers for *bla*$_{TEM}$ are from Monstein, H. J., Ostholm-Balkhed, M. V. Nilsson, M. Nilsson, K. Dornbusch, and L. E. Nilsson. 2007. Multiplex PCR amplification assay for the detection of *bla*$_{SHV}$, *bla*$_{TEM}$, and *bla*$_{CTX-M}$ genes in Enterobacteriaceae. APMIS 115:1400-1408. The remainder of primer pairs were designed for the current study.

All PCR runs were conducted using the following thermal cycling program:

1. Initial heating/denaturation: 95 °C for 4 minutes
2. 30 cycles of the following:
    a. 95 °C, 30 seconds,
    b. 53 °C, 1 minute,
    c. 72 °C, 2 minutes,
3. Final elongation: 72 °C, 4 minutes
4. Cooling and incubation at 4 °C

PCRs were performed using Roche's FastStart Taq PCR kit in total volume of 25 µL according to the manufacturer's instructions. Final concentrations of the components: dNTPs – 200 µM (0.5 µL of 10 mM stock/reaction), each of the primers 200 nM (0.5 µL of 10 µM stock/reaction), enzyme 1.25 U (0.25 µL of 5 U/µL stock/reaction). The quantity of template used was 10 ng/reaction.
