# Supplementary material for: Impact of template denaturation prior to whole genome amplification on gene detection in high GC-content species, Burkholderia mallei and B. pseudomallei
Source: BMC Res Notes. 2024 Mar 12;17:70. doi: 10.1186/s13104-024-06717-8 (PMC10935807; doi:10.1186/s13104-024-06717-8)
Supplement: Supplementary file 3 — Additional file 3. Distribution of yields from WGA; Description—Distribution curves of yields from WGA amplicons from thermally and chemically denatured templates. [file 13104_2024_6717_MOESM3_ESM.pdf]

Additional File 3: Distribution of WGA yields

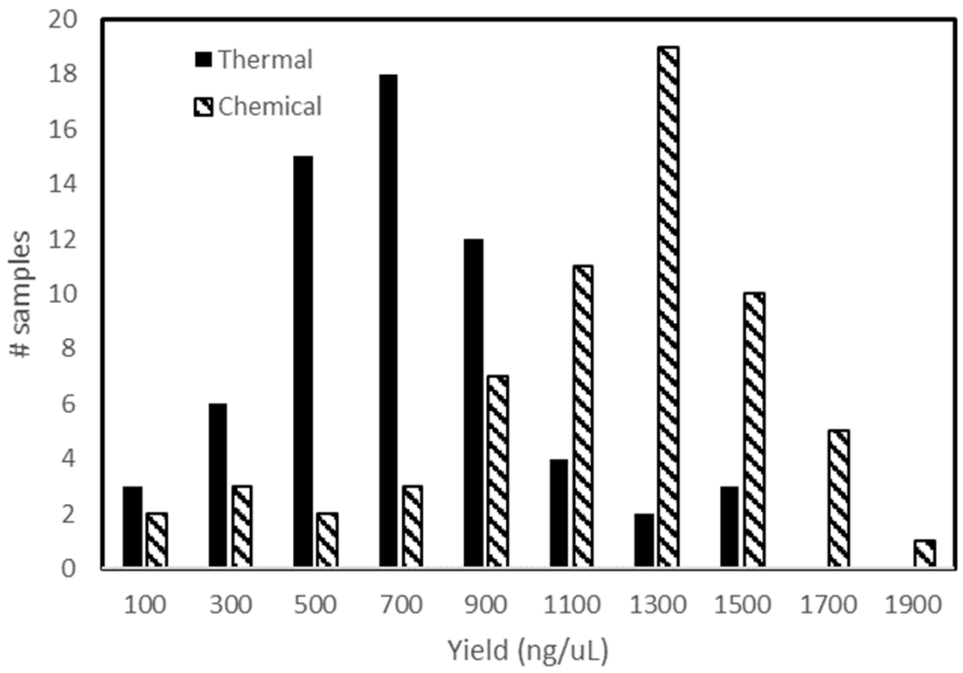

Description: Distribution curves from templates that were thermally (solid bars) or chemically denatured (striped bars); data represent yields from n = 57 matched pairs of UCC preparations + 7 replicate pairs of B. pseudomallei 1026b genomic DNA obtained through BEI Resources, NIAID, NIH
