# Supplementary material for: Impact of template denaturation prior to whole genome amplification on gene detection in high GC-content species, Burkholderia mallei and B. pseudomallei
Source: BMC Res Notes. 2024 Mar 12;17:70. doi: 10.1186/s13104-024-06717-8 (PMC10935807; doi:10.1186/s13104-024-06717-8)
Supplement: Supplementary file 5 — Additional file 5. Putative false-positive (non-BM/BP-derived) genes detected by ARDM; Description—Prevalence of non-BM/BP-derived genes detected by ARDM analysis amongst thermal and chemical samples (Panel A) and distribution of GC contents of non-BM/BP-derived genes detected (Panel B). [file 13104_2024_6717_MOESM5_ESM.pdf]

Additional File 5. Putative false-positive (non-BM/BP-derived) genes detected by ARDM

A		B. mallei		B. pseudomallei	
AMR determinant	GC content (%)	Thermal (n = 22)	Chemical (n = 19)	Thermal (n = 37)	Chemical (n = 34)
aac(3)-ii	66.4		1 (5%)		
aac(6)-ic	64.9		1 (5%)		5 (15%)
aac(6)-iia	58.2				3 (9%)
aac(6)-ik	38.0			1 (3%)	
aadA1/A2	53.6				1 (3%)
aph(3)-iib	70.9				1 (3%)
arr	46.6				4 (12%)
bla_{MIR}	57.9				1 (3%)
bla_{NDM}	61.5				1 (3%)
bla_{OXA-48}	44.5	3 (14%)			
erm42	27.9			4 (11%)	
fexA	42.6			2 (5%)	
floR	58.6	1 (5%)	2 (11%)		1 (3%)
intI3	61.4		1 (5%)		
mphA/ mphK	65.5				2 (6%)
msrA/B/SA	31.2			3 (8%)	
penA-BC	71.4				4 (12%)
vcaM	51.9				4 (13%)
bla_{TEM}	49.1	7 (32%)		12 (32%)	

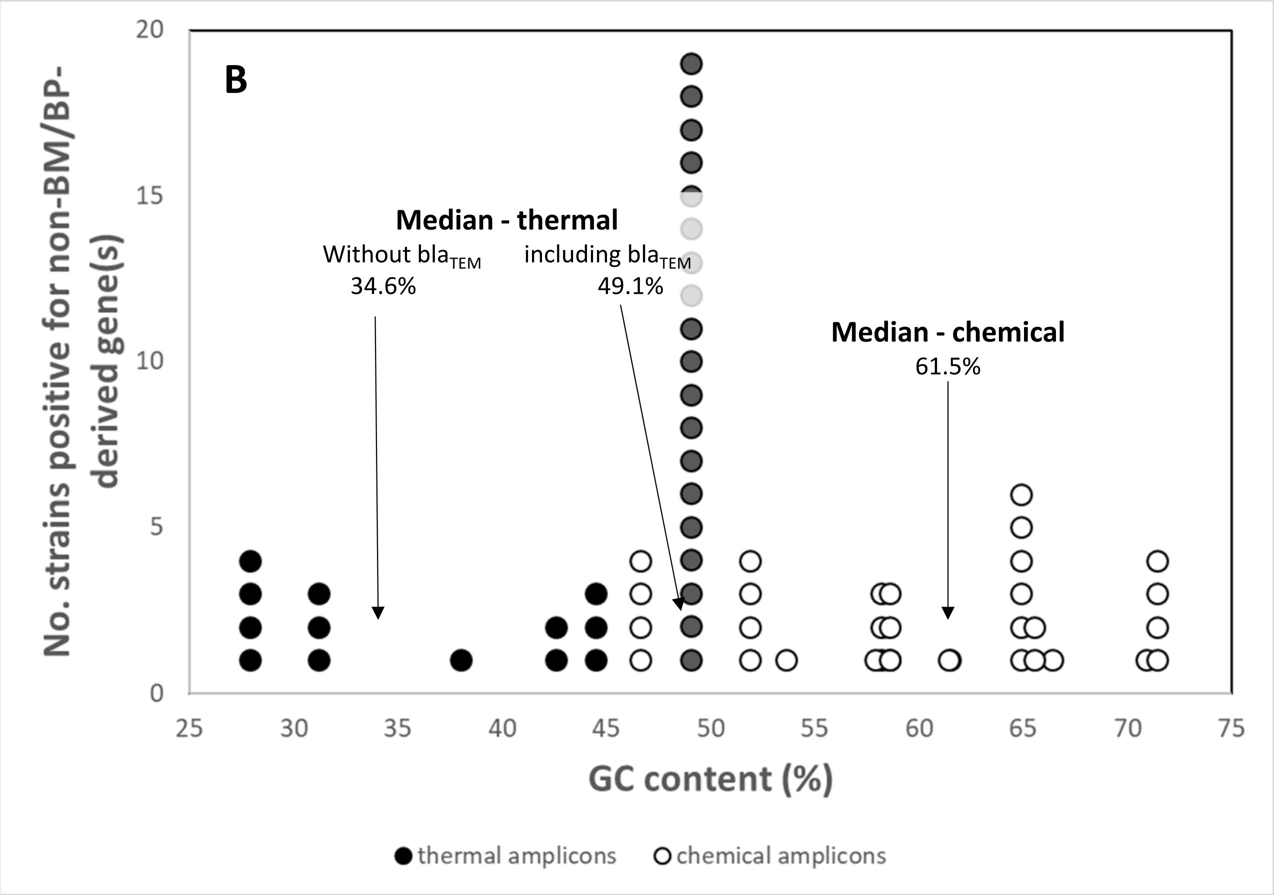

A: Non-BM/BP-derived genes detected in each species, sorted by method for WGA template denaturation
B: Distribution of genes detected in thermal (black circles) and chemical amplicons (white circles) as a function of gene GC content; *bla*$_{TEM}$, shown as dark gray circles, was detected in thermal amplicons only.
